# Is There a Connection between Hyperhomocysteinemia and the Cardiometabolic Syndrome?

**DOI:** 10.3390/biomedicines12061135

**Published:** 2024-05-21

**Authors:** Bogdan Mihai Tarcau, Andra Negru, Timea Claudia Ghitea, Eleonora Marian

**Affiliations:** 1 Doctoral School of Biological and Biomedical Sciences, University of Oradea, 410087 Oradea, Romania; tarcaubogdanmihai@gmail.com; 2 Department of Internal Medicine, Iuliu Hatieganu University of Medicine and Pharmacy, 400347 Cluj-Napoca, Romania; negru_andra09@yahoo.com (A.N.); marian_eleonora@yahoo.com (E.M.); 3 Pharmacy Department, Faculty of Medicine and Pharmacy, University of Oradea, 410087 Oradea, Romania

**Keywords:** hyperhomocysteinemia, metabolic syndrome, obesity, nutrigenetic tests

## Abstract

This study investigates the distribution of hyperhomocysteinemia and cardiovascular metabolic syndrome (SM) among participants, shedding light on their prevalence and co-occurrence within the study cohort. Through an analysis of demographic characteristics and health parameters, including age, gender, and body mass index (BMI), alongside nutritional data, correlations between these factors and health risks are explored. Results reveal a notable prevalence of hyperhomocysteinemia, with 45.3% of participants exhibiting this condition. Furthermore, 31.4% of the cohort does not present hyperhomocysteinemia or SM, while 23.3% shows SM without hyperhomocysteinemia. The study underscores gender-specific dietary recommendations due to significant variations in nutrient intake patterns. Additionally, inverse correlations between health risks like obesity, hypertension, and hypercholesterolemia and nutrient requirements highlight the need for tailored dietary interventions. Age-related changes in nutrient needs and the positive correlation between physical activity levels and certain nutrient demands further emphasize the importance of personalized dietary strategies. Variations in nutrient intake by gender, inverse correlations with health risks, and age-related changes underscore the need for tailored dietary strategies. These findings provide valuable insights for healthcare professionals in developing targeted nutritional interventions to mitigate disease risk and promote overall health and well-being.

## 1. Introduction

Homocysteine, an organic sulfur compound derived from the metabolism of the amino acid methionine, serves pivotal roles in various physiological processes. Its metabolism involves intricate chemical reactions, tightly regulated to maintain body homeostasis. Under normal metabolic efficiency, plasma homocysteine levels fluctuate within a specific range, reflecting a delicate synthesis–catabolism balance [1].

Homocysteine has exhibited a weak association with the presence of metabolic syndrome (SM), while also showing individual associations with some of its components [2,3]. Homocysteine emerges as a promising biomarker for cardiovascular disease, potentially pivotal in linking metabolic syndrome with cardiovascular conditions. Elevated homocysteine levels are thought to inflict damage upon endothelial cells [4]. A recent investigation found that increased levels of insulin can disrupt the processing of homocysteine, resulting in higher concentrations of this compound. In metabolic syndrome, insulin resistance manifests as elevated insulin levels [4,5].

Cysteine, in addition to its crucial role in protein synthesis, assumes pivotal functions in maintaining redox homeostasis, serving as a constituent of the primary antioxidant glutathione and exhibiting potent antioxidant properties independently. It is becoming increasingly evident that events modulated by redox mechanisms play significant roles not only in peripheral tissues but also in the brain, where cysteine disposition is central to these pathways. Moreover, cysteine undergoes diverse post-translational modifications that regulate numerous physiological processes. The dysregulated metabolism of cysteine is linked to various neurodegenerative disorders. Consequently, restoring cysteine balance yields therapeutic advantages [6]. Cysteine occurs naturally in meat, fish, grains, dairy, soybean, and egg products [7].

The metabolic repercussions of homocysteine are linked to diverse health aspects, with imbalances bearing significant consequences [8]. Hyperhomocysteinemia, marked by elevated blood homocysteine levels, can stem from genetic deficiencies, B vitamin deficiencies (e.g., folic acid, vitamin B12), or certain pathologies like hypothyroidism and kidney dysfunction [9].

Of notable concern is its association with vascular disease. Epidemiological studies identify hyperhomocysteinemia as an independent risk factor for conditions such as myocardial infarction, stroke, and atherosclerosis [10]. This linkage, proposed by Kilmer McCully in the 1960s, gained strength through subsequent research demonstrating the increased risk of premature coronary heart disease in individuals with impaired homocysteine metabolism [11].

Homocysteine’s biological significance extends to its interactions with folic acid, vitamin B12, and related metabolic enzymes, further complicated by genetic polymorphisms, hormonal influences, and other factors. Hence, delving into homocysteine’s role in human health is crucial for comprehending disease mechanisms and crafting prevention and treatment approaches [12,13].

This paper aims to track genetic variations of hyperhomocysteinemia in individuals with metabolic syndrome. Objectives include investigating prevalence and severity, analyzing correlations with metabolic syndrome markers, and assessing the impact on metabolic complication risks.

## 2. Materials and Methods

We conducted a prospective study involving patients seeking dietary consultation, undergoing nutrigenetic testing, and providing anthropometric data and physical activity levels. Written consent was obtained from participants between January 2019 and December 2023.

Inclusion Criteria:

Patients aged 18 or above undergoing nutrigenetic testing, willing to participate, and aiming to improve quality of life through diet and lifestyle changes.

Exclusion Criteria:

Patients under 18 years old or those unwilling to participate.

The patients were selected from the nutrition clinic based on different degrees of obesity, according to the inclusion criteria. They were divided into groups based on the presence or absence of metabolic syndrome (MS) or hyperhomocysteinemia. A total of 86 patients were divided into three research groups based on metabolic syndrome (SM) presence. 39 individuals (45.3%) exhibited hyperhomocysteinemia (group HH), 27 (31.4%) had neither hyperhomocysteinemia nor cardiovascular metabolic syndrome (group HH without SM, i.e., control group), and 20 (23.3%) had cardiovascular metabolic syndrome without hyperhomocysteinemia (group HH with SM).

### 2.1. Anamnesis and Objective Examination

Through anamnesis and objective examination, data were collected, including age, sex, height, weight for BMI determination, and physical activity levels categorized into four groups.

### 2.2. Nutrigenetic Testing

The Advanced NutriGenetx (Rainbow Court, Cary, NC, USA) test provides validated genetic information on nutrient requirements for healthy adults, pregnant women, metabolic risks, rare genetic diseases, pharmacogenetic tests, and nutrigenetic management for various nutrients and chronic metabolic diseases (i.e., Gene *MTHFR*, Marker rs1801133 and Gene *TCF7L2*, Marker rs12255372). This test is a genetic test that defines individual nutritional needs based on the analysis of haplotypes, gene interactions and multigene algorithms.

### 2.3. Nutrigenetic Management of Physically Active Individuals or Athletes

This genetic analysis entails estimating your potential for engaging in certain types of physical exertion and the potential impact this exertion may have on your metabolism. The information obtained can be utilized by professionals in the field, including doctors, nutritionists, and fitness specialists, to make informed decisions regarding necessary nutritional adjustments and beneficial physical activities aimed at achieving optimal weight. The analysis also encompasses genetic variations that influence the optimal nutrient requirements for adults, thereby reducing the risk of metabolic diseases resulting from a diet that does not align with individual genetic makeup. Additionally, genetic variations are examined that, in the presence of diseases or unhealthy lifestyles, may lead to the development of complications in the absence of proper nutrition. Furthermore, a genetic score for non-alcoholic hepatosteatosis is provided for the purpose of appropriate nutritional management. Addresses muscle strength, cardiorespiratory function, adiposity, body weight, glucose and insulin metabolism, and lipid and lipoprotein metabolism.

### 2.4. Statistical Analysis

Data analysis utilized IBM’s Statistical Product and Service Solutions (SPSS version 20), NY, USA. Demographic variables, procedure frequency, and cost data from the medical office were evaluated across two time points and study groups to identify trends. Means, frequency ranges, standard deviations, and statistical significance were calculated using Student’s *t*-test and the chi-square test. The Bravais–Pearson correlation coefficient assessed variable relationships. Statistical significance was defined as *p* < 0.05, with *p* < 0.01 indicating high significance. Post hoc analysis (Holm–Bonferroni) further scrutinized group differences.

## 3. Results

**Figure 1:**
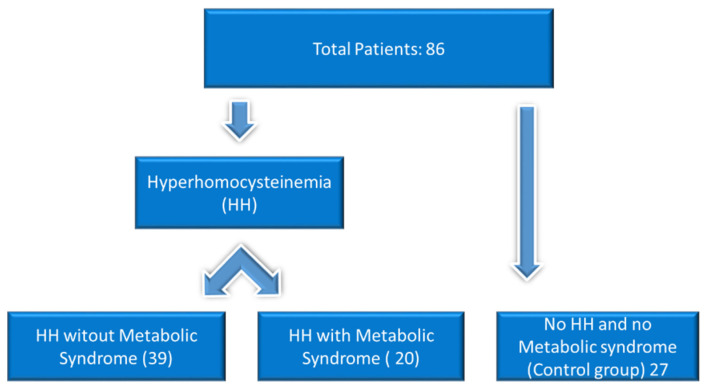
Flow chart.

The demographic description of the study patients is as follows, presented and distributed in parametric groups. The majority in the urban environment (62.79%). The count for Masculine is 35, constituting 40.7% of the total, while Feminine has a count of 51, making up 59.3% of the total. Regarding the variable Age, the mean age is 43.37 with a standard deviation of 12.71. The flow chart is presented in Figure 1.

### 3.1. The Study of the Parameters According to the Research Cohort

**Figure 2:**
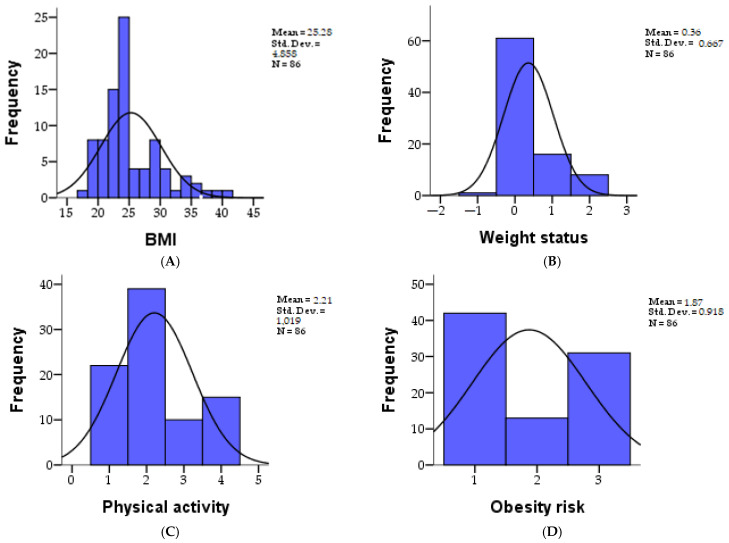

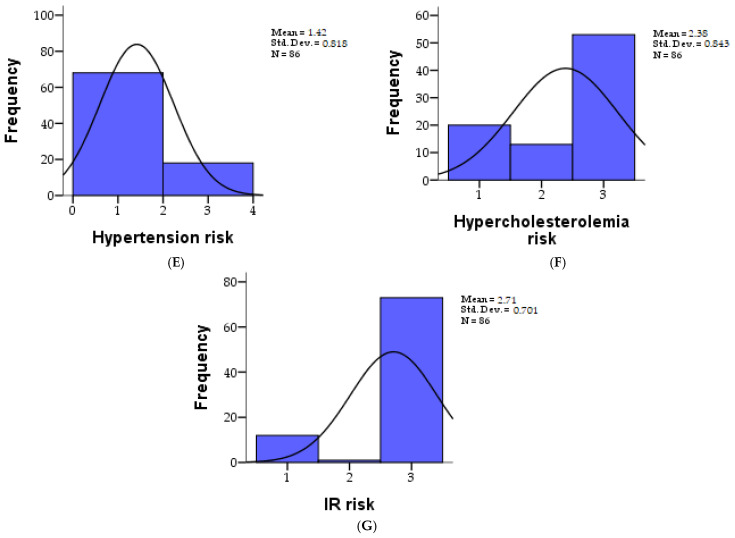
Graphic presentation of parameters as BMI (**A**), weight status (**B**), physical activity (**C**), obesity risk (**D**), hypertension risk (**E**), hypercholesterolemia risk (**F**) and insulin resistance (IR) risk (**G**), at the level of the study cohort.

The mean for Masculine individuals in terms of IMC (Body Mass Index) is 25.31, with a corresponding count. For Feminine individuals, the mean IMC is 25.26. Under the category of Ponderal Status, individuals are classified as subponderal, normoponderal, supraponderal, Obese I, Obese II, and Obese III. The percentage distribution across these categories differs between Masculine and Feminine individuals. Similarly, variables like Physical Activity, Obesity Risk, Hypertension Risk, Hypercholesterolemia Risk, and Insulin Resistance Risk are also examined. Each of these variables is categorized into different risk levels (Low, Medium, High), and the distribution of individuals across these categories is provided for both Masculine and Feminine groups. The descriptive statistics of the research parameters at the cohort level can be found in Table 1, and the distribution of values in Figure 2.

The nutritional data are divided by sex (masculine and feminine) and include various nutrients along with their recommended daily intake levels. Here is a description based on the provided values:

For males: Zinc necessary (mg/day): The mean intake is 13.71 mg/day. Magnesium necessary (mg/day): The mean intake is 504.86 mg/day. Folic Acid necessary (UEF-5-MTHF): For 65.7% of males, the intake ranges from 400 UEF-5-MTHF, while for 34.3%, it is exactly 400 UEF. Vitamin B2 necessary (mg/day): The mean intake is 2.49 mg/day. Vitamin B3 necessary (mg/day): The mean intake is 23.77 mg/day. Vitamin B6 necessary (mg/day): The mean intake is 1.45 mg/day. Vitamin B12 necessary (µg/day): The mean intake is 4.80 µg/day. Betaine necessary (mg/day): The mean intake is 200.00 mg/day. N6:N3 ratio: For 100% of males, the ratio is less than 4, indicating a desirable intake.

For females: Zinc necessary (mg/day): The mean intake is 10.90 mg/day. Magnesium necessary (mg/day): The mean intake is 387.84 mg/day. Folic Acid necessary (UEF-5-MTHF): For 72.5% of females, the intake ranges from 400 UEF-5-MTHF, while for 27.5%, it is exactly 400 UEF. Vitamin B2 necessary (mg/day): The mean intake is 2.21 mg/day. Vitamin B3 necessary (mg/day): The mean intake is 20.51 mg/day. Vitamin B6 necessary (mg/day): The mean intake is 1.35 mg/day. Vitamin B12 necessary (µg/day): The mean intake is 4.79 µg/day. Betaine necessary (mg/day): The mean intake is 200.00 mg/day. N6:N3 ratio: For 15.7% of females, the ratio is less than 4, while for 84.3%, it is less than 10, indicating a desirable intake. The descriptive statistics of the research parameters of the nutrition data at the cohort level can be found in Table 2, and the distribution of values in Figure 2.

### 3.2. The Study of the Parameters According to the Research Groups

Table 3 presents data on parameters including hyperhomocysteinemia status and cardiovascular metabolic syndrome (SM) in relation to sex, age, and body mass index (IMC). Regarding hyperhomocysteinemia, among males, 17.4% do not have hyperhomocysteinemia or SM, while 16.3% have no hyperhomocysteinemia but have SM. Among females, 27.9% do not have hyperhomocysteinemia or SM, 15.1% have no hyperhomocysteinemia but have SM, and 16.3% have both conditions. The mean age for those with hyperhomocysteinemia without SM is 43.56 ± 11.73, for those without hyperhomocysteinemia and SM it is 40.00 ± 12.17, and for those with hyperhomocysteinemia and SM it is 47.55 ± 14.51. Similarly, the mean body mass index (IMC) for those with hyperhomocysteinemia without SM is 25.42 ± 4.26, for those without hyperhomocysteinemia and SM it is 23.06 ± 2.69, and for those with hyperhomocysteinemia and SM it is 27.99 ± 6.68.

Table 4 provides a comprehensive overview of various nutritional parameters categorized by the presence or absence of hyperhomocysteinemia and cardiovascular metabolic syndrome (SM) across different sexes. It includes mean values, counts, and percentages for each parameter. For instance, for individuals without hyperhomocysteinemia and SM, the mean intake of zinc is 13.14 mg/day for males and 9.08 mg/day for females. Similarly, the mean intake of magnesium is 503.57 mg/day for males and 386.92 mg/day for females in the same group. Furthermore, the table depicts the distribution of folic acid intake, with 66.7% of males and 83.3% of females consuming the recommended dosage of 400 UEF-5-MTHF in the absence of hyperhomocysteinemia and SM. Additionally, it presents the N6:N3 ratio, where 100% of males and females have a ratio of less than 4 in the absence of hyperhomocysteinemia, indicating a desirable intake.

**Figure 3:**
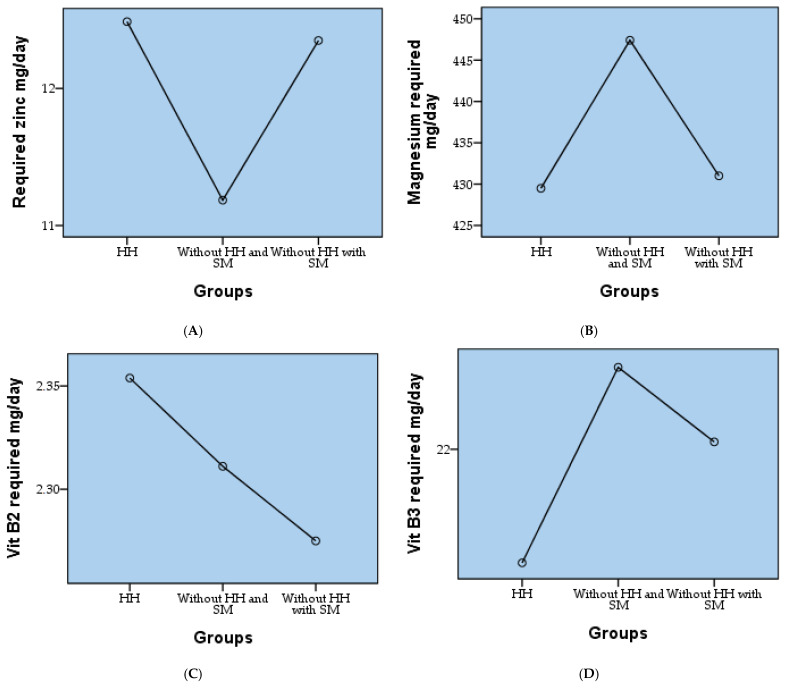

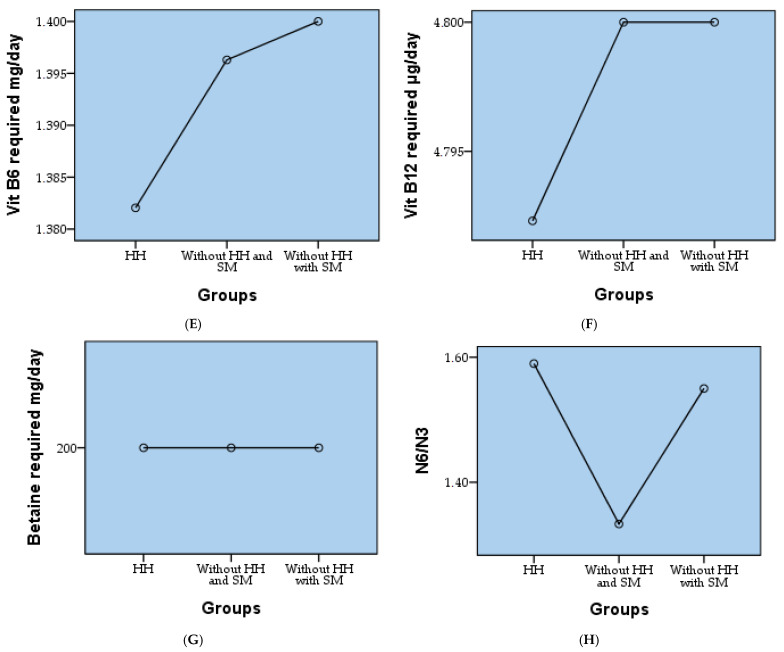
The variation in average values of nutritional parameters across different lots as zinc (**A**), magnesium (**B**), vitamin B2 (**C**), vitamin B3 (**D**), vitamin B6 (**E**), vitamin B12 (**F**), betaine (**G**) and N6/N3 (**H**).

Figure 3 illustrates the variation in average values of nutritional parameters across different lots categorized by the presence or absence of hyperhomocysteinemia and cardiovascular metabolic syndrome (SM). Comparing the mean values across the three lots, notable differences can be observed. For instance, concerning zinc intake, the mean value is 12.49 mg/day for individuals with hyperhomocysteinemia, with a statistically significant difference (*p* = 0.001) from 11.19 mg/day for those without hyperhomocysteinemia but without SM, and with statistically significant difference from (*p* = 0.017) 12.35 mg/day for individuals without hyperhomocysteinemia but with SM. A statistically insignificant difference was observed between HH and Without HH with SM. Similarly, for magnesium intake, the mean values are 429.49 mg/day, with a statistically significant difference (*p* = 0.001) from 447.41 mg/day, and with a statistically significant difference (0.007) from 431.00 mg/day for each respective lot. A statistically insignificant difference was observed between HH and Without HH with SM. Notably, there is a fluctuation in the intake of folic acid, with individuals consuming 400 UEF-5-MTHF having a mean intake of 30 and 13 for lots with and without hyperhomocysteinemia, respectively, while for those with SM, the mean intake is 17. A statistically significant difference was observed between all groups (*p* = 0.001). This trend is consistent across several other nutrients such as Vitamin B2, B3, and B6, where slight differences in mean intake are observed among the three lots. Additionally, the N6:N3 ratio exhibits variations across lots, with differing proportions of individuals meeting the desirable intake level, particularly evident in the <4 category, where the mean count differs across lots. Significant differences were also recorded in the case of B3 and B6 between each group (*p* < 0.05). Overall, the table underscores the significance of considering the lot classification when analyzing average nutritional values, as it highlights distinct consumption patterns among individuals with varying health conditions. In the case of betaine, no statistically significant differences were recorded, with all 3 groups having similar values.

### 3.3. Correlations

Statistically significant positive correlations were recorded between age and the need for vitamin B6, between physical activity and the need for zinc and magnesium, respectively, and between the risk of hypertension and the need for zinc (Table 5). This correlation reflects a direct proportional relationship, as one of the values increases, the other also increases. As age increases, so does the need for vitamin B6, increasing physical activity leads to an increased need for zinc and magnesium, and as the risk of hypertension increases, so does the need for zinc.

Inversely proportional, statistically significant correlations were recorded between:

- gender and the need for zinc, magnesium, vitamin B2, vitamin B3 and vitamin B6, respectively
- age and folic acid
- weight status and zinc requirement
- risk of obesity and the need for zinc, magnesium, vitamin B2, vitamin B3
- the risk of hypertension and the need for folic acid and vitamin B3
- the risk of hypercholesterolemia and the need for folic acid
- N6/N3 in relation to zinc, magnesium, vitamin B2, B3, and B6 requirements.

As the risk of obesity, hypertension, and hypercholesterolemia increases, the need for nutrients decreases. This ratio is also reflected in those with the N6/N3 ratio, the higher the ratio, the lower the nutrient requirement. These inversely proportional relationships can be explained by the fact that health risks reduce metabolic activity and aggravate the metabolic syndrome.

## 4. Discussion

Marc Lalonde’s theory encompasses a new perspective on the health of Canadians, identifying genetics, environment, personal lifestyle, and medical care as equally important factors in both personal and population health. Based on the concrete description of health problems induced by obesity, high-fat intake, high-carbohydrate intake, or lack of exercise, Lalonde formulated concrete proposals to address these issues within the context of health problems in Canada. Health promotion has assumed a crucial role in smoking reduction and dietary modification to address the pandemics of lung cancer and heart disease [14]. This theory can be regarded as the foundation of numerous ideas promoting healthy lifestyles. 

Homocysteine, a sulfurized amino acid resulting from methionine metabolism, has garnered significant interest in medical research due to its potential implications in vascular pathology, cognitive function, and other aspects of human health. While the human body requires homocysteine for certain essential metabolic processes, elevated concentrations of this compound have been associated with various conditions, including cardiovascular disease, cognitive dysfunction, and pregnancy complications [15,16]. The increase in homocysteinemia is directly correlated with the calcification of coronary and extracoronary vessels, according to a study published by Karger in 2020 [17]. Luzzi (2022) also observed a link between cognitive decline and hyperhomocysteinemia, specifically in patients with dementia [18]. A high level of hyperhomocysteinemia was recorded in those with cognitive decline. In the current study, we examine the associated diseases in people with hyperhomocysteinemia. Another study that investigated homocysteine levels in the context of reproductive health found that low homocysteine levels are associated with reproductive health, while high levels can negatively affect it [19].

Several causes of hyperhomocysteinemia have been identified in studies, including genetic factors such as polymorphisms in enzymes involved in homocysteine metabolism, B vitamin deficiency, kidney disease, and hypothyroidism [20]. Research has shown that various polymorphisms in genes within the homocysteine–methionine pathway lead to hyperhomocysteinemia, indicating that these genetic variations might contribute to numerous common multifactorial disorders prevalent in the general population [21]. Hyperhomocysteinemia has also been observed in the context of conditions such as diabetes mellitus, and various drugs such as anti-Parkinson’s and anti-epileptic drugs can influence homocysteine levels in the blood. In our study, gender-specific dietary recommendations were noted to be crucial [22]. It has been suggested that N-acetylcysteine (NAC) disrupts the disulfide bonds in Lp(a) as well as between homocysteine and plasma proteins, thus leading to a significant decrease in homocysteine levels [23]. In this study, only nutritional intervention was employed, aiming to achieve results comparable to diet therapy alone.

The role of homocysteine in vascular pathology has been extensively studied, revealing that elevated homocysteine levels are linked to increased risks of cardiovascular disease [24], cerebrovascular [25], vitamin D deficiency [26,27] and peripheral vascular disease [28], primarily due to heightened inflammation. Various epidemiological studies have noted this association, prompting intensive research into the precise mechanisms by which homocysteine contributes to these conditions, including changes to endothelial cell surfaces, platelet activation, and peroxide-induced oxidative damage [29]. 

Following the research parameters obesity, hypertension and hypercholesterolemia have been observed to have an inverse impact on nutrient requirements [30,31,32]. 

The involvement of endothelial and inducible NOS isoforms in the collagen/elastin switch has been established. Our findings indicate that elevated inducible NOS activity is a crucial element in the collagen/elastin switching process mediated by hyperhomocysteinemia [33].

Beyond its vascular implications, homocysteine has also been examined in relation to cognitive function and the risk of dementia in older adults. Research indicates that hyperhomocysteinemia can impair cognitive function, with elevated homocysteine levels being associated with a higher risk of Alzheimer’s disease and dementia. Additionally, higher physical activity levels correlate with increased demands for nutrients [34,35].

Homocysteine also plays a role in reproductive health, suspected as a risk factor for pregnancy complications and birth defects [36]. Supplementation with B vitamins, particularly folic acid, has been investigated as a strategy to reduce homocysteine levels and prevent these complications. There has been a positive relationship between age and the need for certain nutrients such as vitamin B6 [37,38].

Due to its role in maintaining cellular redox balance, alterations in cysteine levels trigger various compensatory or corrective responses. Homocysteine is implicated in the pathogenesis of cardiovascular disease through multiple mechanisms, including its detrimental effects on vascular endothelium and smooth muscle cells, resulting in changes in subclinical arterial structure and function [11]. The present study examined the association between hyperhomocysteinemia and the prevalence of cardiovascular metabolic syndrome. The identification of hyperhomocysteinemia was deemed significantly important, particularly as metabolic issues become increasingly pronounced.

Interventional studies assessing the efficacy of B vitamin supplementation in reducing the vascular or cognitive risks associated with hyperhomocysteinemia have yielded conflicting results [39]. While some studies have reported benefits in lowering homocysteine levels through supplementation, others have not demonstrated significant advantages in preventing major vascular events or cognitive decline [40,41].

The study of the genotype of hyperhomocysteinemia is a relatively new and quite expensive method, which can be considered one of the limitations of the study. The information collected from patients in this study, which correlated with existing diseases, was gathered at a medical center from patients who already had health problems (MS), presenting another limitation. Thus, we propose expanding the studies to include a larger cohort of individuals and conducting a more comprehensive analysis of incidents and associated risks.

Despite the controversy, homocysteine remains the subject of intense investigation to clarify its role in vascular and neurodegenerative pathology. This discussion explores various aspects of the complex relationship between homocysteine, its metabolism, and its impact on human health, highlighting the challenges and uncertainties in fully understanding this biological phenomenon.

The global impact of these conclusions highlights the need for gender-specific dietary recommendations due to significant differences in nutritional intake patterns. Tailored dietary interventions are essential, as conditions like obesity, hypertension, and hypercholesterolemia affect nutrient requirements. Nutritional needs evolve with age, particularly for nutrients like vitamin B6, and higher physical activity levels increase demands for nutrients such as zinc and magnesium.

Understanding the relationship between nutrient intake, health risks, and metabolic conditions allows for targeted nutritional interventions to improve disease management and overall health. Addressing hyperhomocysteinemia with a diet rich in folic acid and cyanocobalamin can reduce cardiometabolic risks and enhance the quality of life for affected patients. These insights promote a personalized approach to nutrition, aiming to optimize health outcomes globally.

## 5. Conclusions

Gender-specific dietary recommendations are crucial due to significant variations in nutritional intake patterns between genders.

Health conditions like obesity, hypertension, and hypercholesterolemia inversely impact nutrient requirements, highlighting the need for tailored dietary interventions.

Nutritional needs may evolve with age, as seen in the positive correlation between age and the need for certain nutrients like vitamin B6.

Higher physical activity levels correlate with increased demands for nutrients like zinc and magnesium to support energy metabolism and muscle function.

Understanding the relationship between nutrient intake, health risks, and metabolic conditions informs targeted nutritional interventions for disease management and overall health promotion.

Identifying hyperhomocysteinemia emphasizes the importance of implementing specialized anti-inflammatory dietary therapy in managing a healthy lifestyle. A specific and personalized diet focuses on high intake of folic acid and cyanocobalamin, consequently leading to a notable reduction in cardiometabolic risks. Such measures aim to improve the quality of life of patients diagnosed with hyperhomocysteinemia.

## Figures and Tables

**Table 1:** The descriptive statistics of the metabolic parameters at the cohort level.

Parameters	N	Mean	SD	Skewness	Kurtosis	Minim	Maxim	Percentiles		
								25	50	75
BMI	86	25.27	4.857	1.180	1.263	17.94	41.66	22.2075	24.0900	28.0550
Weight status	86	0.36	0.667	1.382	1.031	−1.00	2.00	0.0000	0.0000	1.0000
Physical activity	86	2.20	1.018	0.590	−0.704	1.00	4.00	1.0000	2.0000	3.0000
Obesity risk	86	1.87	0.917	0.259	−1.784	1.00	3.00	1.0000	2.0000	3.0000
Hypertension risk	86	1.41	0.818	1.455	0.118	1.00	3.00	1.0000	1.0000	1.0000
Risk of hypercholesterolemia	86	2.38	0.842	−0.832	−1.073	1.00	3.00	2.0000	3.0000	3.0000
IR risk	86	2.70	0.700	−2.048	2.297	1.00	3.00	3.0000	3.0000	3.0000

BMI = body mass index, IR = insulin resistance, N = number of patients.

**Table 2:** The descriptive statistics of the nutritional parameters at the cohort level.

Parameters	N	Mean	SD	Skewness	Kurtosis	Minim	Maxim	Percentiles		
								25	50	75
Required zinc mg/day	86	12.04	3.300	0.889	0.845	8.00	22.00	10.0000	11.0000	14.0000
Magnesium required mg/day		435.46	61.675	0.066	−1.025	250.00	510.00	390.0000	390.0000	510.0000
Folic acid required day		1.30	0.461	0.876	−1.262	1.00	2.00	1.0000	1.0000	2.0000
Vit B2 required mg/day		2.32	0.275	−1.498	4.578	1.30	2.60	2.2000	2.2000	2.6000
Vit B3 required mg/day		21.83	2.365	−1.635	3.759	14.00	24.00	21.0000	21.0000	24.0000
Vit B6 required mg/day		1.39	0.149	1.294	0.072	1.30	1.70	1.3000	1.3000	1.5000
Vit B12 required µg/day		4.79	0.032	−9.274	86.000	4.50	4.80	4.8000	4.8000	4.8000
Betaine required mg/day		200.00	0.000	-	-	200.00	200.00	200.0000	200.0000	200.0000
N6/N3		1.50	0.502	0.000	−2.048	1.00	2.00	1.0000	1.5000	2.0000

N = number of patients, SD = standard deviation, Vit = vitamin, N6/N3 = omega6/omega3.

**Table 3:** Metabolic parameters by groups.

Parameters		Groups					
		HH		Without HH and SM		Without HH with SM	
		Count	%	Count	%	Count	%
BMI (Mean ± SD)		25.42 ± 4.26		23.06 ± 2.69		27.99 ± 6.68	
Weight status	underweight	0	0.0%	0	0.0%	1	1.2%
	normal	26	30.2%	25	29.1%	10	11.6%
	overweight	8	9.3%	1	1.2%	7	8.1%
	obesity I	5	5.8%	1	1.2%	2	2.3%
	obesity II	0	0.0%	0	0.0%	0	0.0%
	obesity III	0	0.0%	0	0.0%	0	0.0%
Physical activity (Mean ± SD)		2.21 ± 1.03		2.41 ± 0.97		1.95 ± 1.05	
Obesity risk	low	12	14.0%	21	24.4%	9	10.5%
	medium	13	15.1%	0	0.0%	0	0.0%
	high	14	16.3%	6	7.0%	11	12.8%
Hypertension risk	low	29	33.7%	27	31.4%	12	14.0%
	medium	0	0.0%	0	0.0%	0	0.0%
	high	10	11.6%	0	0.0%	8	9.3%
Risk of hypercholesterolemia	low	2	2.3%	15	17.4%	3	3.5%
	medium	12	14.0%	0	0.0%	1	1.2%
	high	25	29.1%	12	14.0%	16	18.6%
IR risk	low	4	4.7%	8	9.3%	0	0.0%
	medium	1	1.2%	0	0.0%	0	0.0%
	high	34	39.5%	19	22.1%	20	23.3%

N = number of patients, SD = standard deviation, IR = insulin resistance.

**Table 4:** Nutritional parameters by groups.

Parameters		Groups					
		HH		Without HH and SM		Without HH with SM	
		Count	%	Count	%	Count	%
Required zinc mg/day (Mean ± SD)		12.49 ± 3.42		11.19 ± 2.40		12.35 ± 3.99	
Magnesium required mg/day (Mean ± SD)		429.49 ± 64.11		447.41 ± 60.17		431.00 ± 59.55	
Folic acid required day	400 UEF-5-MTHF	30	34.9%	13	15.1%	17	19.8%
	400	9	10.5%	14	16.3%	3	3.5%
Vit B2 required mg/day (Mean ± SD)		2.35 ± 0.20		2.31 ± 0.35		2.28 ± 0.30	
Vit B3 required mg/day (Mean ± SD)		21.23 ± 3.01		22.56 ± 1.53		22.05 ± 1.47	
Vit B6 required mg/day (Mean ± SD)		1.38 ± 0.14		1.40 ± 0.16		1.40 ± 0.15	
Vit B12 required µg/day (Mean ± SD)		4.79 ± 0.05		4.80 ± 0.00		4.80 ± 0.00	
Betaine required mg/day		200.00 ± 0.00		200.00 ± 0.00		200.00 ± 0.00	
N6/N3	<4	16	18.6%	18	20.9%	9	10.5%
	<10	23	26.7%	9	10.5%	11	12.8%

N = number of patients, SD = standard deviation, Vit = vitamin, N6/N3 = omega6/omega3.

**Table 5:** Pearson correlation regarding research parameters.

Pearson Correlation		Sex	Age	Weight Status	Physical Activity	Obesity Risk	Hypertension Risk	Hypercholesterolemia Risk	N6/N3
Required zinc mg/day	r	−0.421 **	0.076	−0.221 *	0.228 *	−0.282 **	0.672 **	0.167	−0.298 **
	*p*	0.000	0.489	0.040	0.035	0.009	0.000	0.124	0.005
Vit B3 required mg/day	r	−0.681 **	0.126	−0.015	0.131	−0.394 **	−0.268 *	−0.045	−0.633 **
	*p*	0.000	0.246	0.894	0.228	0.000	0.013	0.681	0.000
Magnesium required mg/day	r	−0.938 **	0.106	−0.129	0.219 *	−0.624 **	−0.186	−0.011	−0.825 **
	*p*	0.000	0.329	0.238	0.042	0.000	0.087	0.917	0.000
Folic acid required day	r	−0.073	−0.234 *	−0.129	−0.061	0.065	−0.276 **	−0.302 **	−0.101
	*p*	0.504	0.030	0.237	0.577	0.555	0.010	0.005	0.354
Vit B2 required mg/day	r	−0.504 **	0.077	−0.038	0.139	−0.315 **	−0.021	0.146	−0.447 **
	*p*	0.000	0.479	0.732	0.203	0.003	0.850	0.180	0.000
Vit B6 required mg/day	r	−0.323 **	0.647 **	0.046	−0.064	−0.138	−0.083	0.038	−0.235 *
	*p*	0.002	0.000	0.675	0.556	0.206	0.446	0.728	0.029
Vit B12 required µg/day	r	−0.090	−0.074	0.059	0.129	0.104	0.056	−0.080	−0.108
	*p*	0.411	0.498	0.590	0.235	0.342	0.610	0.465	0.320
N		86							

r = coefficient Pearson, *p* = statistically signification, N = number of patients, N6/N3 = omega6/omega3, * = Correlation is significant at the 0.05 level (2-tailed), ** = Correlation is significant at the 0.01 level (2-tailed).

## Data Availability

All the data processed in this article are part of the research for a doctoral thesis, being archived in the aesthetic medical office, where the interventions were performed.
